# Serum metabolomic signatures of fatty acid oxidation defects differentiate host-response subphenotypes of acute respiratory distress syndrome

**DOI:** 10.1186/s12931-023-02447-w

**Published:** 2023-05-20

**Authors:** Tomeka L. Suber, Stacy G. Wendell, Steven J. Mullett, Benjamin Zuchelkowski, William Bain, Georgios D. Kitsios, Bryan J. McVerry, Prabir Ray, Anuradha Ray, Rama K. Mallampalli, Yingze Zhang, Faraaz Shah, Seyed Mehdi Nouraie, Janet S. Lee

**Affiliations:** 1 Division of Pulmonary, Allergy, Critical Care, and Sleep Medicine, Department of Medicine, Montefiore Hospital, University of Pittsburgh School of Medicine, NW 628, 3459 Fifth Avenue, Pittsburgh, PA 15213 USA; 2 Acute Lung Injury Center of Excellence, Department of Medicine, University of Pittsburgh School of Medicine, Pittsburgh, PA USA; 3 Department of Pharmacology and Chemical Biology, University of Pittsburgh School of Medicine, Pittsburgh, PA USA; 4 Veterans Affairs Pittsburgh Healthcare System, Pittsburgh, PA USA; 5 Department of Internal Medicine, The Ohio State University Wexner Medical Center, Columbus, OH USA; 6 Division of Pulmonary and Critical Care Medicine, Department of Medicine, Washington University at St. Louis, St. Louis, MO USA

**Keywords:** Metabolomics, Acute respiratory distress syndrome, Subphenotypes, Acylcarnitines, Fatty acid oxidation

## Abstract

**Background:**

Fatty acid oxidation (FAO) defects have been implicated in experimental models of acute lung injury and associated with poor outcomes in critical illness. In this study, we examined acylcarnitine profiles and 3-methylhistidine as markers of FAO defects and skeletal muscle catabolism, respectively, in patients with acute respiratory failure. We determined whether these metabolites were associated with host-response ARDS subphenotypes, inflammatory biomarkers, and clinical outcomes in acute respiratory failure.

**Methods:**

In a nested case–control cohort study, we performed targeted analysis of serum metabolites of patients intubated for airway protection (airway controls), Class 1 (hypoinflammatory), and Class 2 (hyperinflammatory) ARDS patients (N = 50 per group) during early initiation of mechanical ventilation. Relative amounts were quantified by liquid chromatography high resolution mass spectrometry using isotope-labeled standards and analyzed with plasma biomarkers and clinical data.

**Results:**

Of the acylcarnitines analyzed, octanoylcarnitine levels were twofold increased in Class 2 ARDS relative to Class 1 ARDS or airway controls (*P* = 0.0004 and < 0.0001, respectively) and was positively associated with Class 2 by quantile g-computation analysis (*P* = 0.004). In addition, acetylcarnitine and 3-methylhistidine were increased in Class 2 relative to Class 1 and positively correlated with inflammatory biomarkers. In all patients within the study with acute respiratory failure, increased 3-methylhistidine was observed in non-survivors at 30 days (*P* = 0.0018), while octanoylcarnitine was increased in patients requiring vasopressor support but not in non-survivors (*P* = 0.0001 and *P* = 0.28, respectively).

**Conclusions:**

This study demonstrates that increased levels of acetylcarnitine, octanoylcarnitine, and 3-methylhistidine distinguish Class 2 from Class 1 ARDS patients and airway controls. Octanoylcarnitine and 3-methylhistidine were associated with poor outcomes in patients with acute respiratory failure across the cohort independent of etiology or host-response subphenotype. These findings suggest a role for serum metabolites as biomarkers in ARDS and poor outcomes in critically ill patients early in the clinical course.

**Supplementary Information:**

The online version contains supplementary material available at 10.1186/s12931-023-02447-w.

## Background

The acute respiratory distress syndrome (ARDS) continues to have high mortality and long-term morbidity burden in survivors [1, 2], a global health challenge further exacerbated by the COVID-19 pandemic [3, 4]. Despite decades of research, effective therapeutic options remain limited to supportive care such as lung-protective mechanical ventilation and treatment of the underlying etiology [5]. Clinical trials in ARDS have largely yielded negative results [6], as one of the significant challenges in the design and interpretation of studies is the heterogeneity of the ARDS population. Well-validated latent class analysis studies and subsequent parsimonious models incorporate clinical characteristics and biomarkers to define two major subphenotypes–hypoinflammatory (herein described as Class 1) and hyperinflammatory (Class 2) [7, 8]. Class 2 is associated with increased inflammatory biomarkers such as soluble tumor necrosis factor receptor 1 (TNFR1) and receptor of advanced glycation end products (RAGE), increased incidence of acute kidney injury, longer duration of mechanical ventilation, and increased mortality in multiple large cohorts [7, 9]. Further subphenotyping using genomics, transcriptomics, and metabolomics data may identify subpopulations of ARDS patients likely to respond to targeted therapies early in their clinical course [10]. Of these, metabolomics studies remain in the earliest stages of development with technical limitations given cost, biological variability, and lack of standardization across samples in some published studies [11–14].

Metabolic derangements and reprogramming occur in ARDS and may provide clues to pathogenesis [15]. One of the earliest studies to examine this question characterized metabolites in pulmonary edema fluid within a cohort of 23 ARDS patients, six of whom exhibited hypermetabolic characteristics and increased mortality, though this study did not include serum biomarker data and preceded current subphenotype classification [16]. In a larger study, two metabolic endotypes were predictive of mortality with profiles differing between serum and mini-bronchoalveolar fluid samples but were not related to host-response ARDS subphenotypes [17]. Most recently, fatty acid oxidation defects were associated with hyperinflammatory ARDS within a sepsis cohort [18]. While few studies have examined metabolomics in the context of host-response subphenotypes in ARDS, the hypo- and hyperinflammatory designations may not fully capture the complexities of these subphenotypes, and few have examined how serum metabolites may differentiate these groups during acute clinical deterioration independent of the etiology of ARDS or acute respiratory failure [12].

Fatty acid oxidation (FAO), also known as β-oxidation, generates acetyl-CoA from fatty acids for entry into the tricarboxylic acid (TCA) cycle for energy production in the form of ATP [19, 20]. Carnitine shuttles acylated fatty acids as acylcarnitines from the cytosol and ultimately to the mitochondrial matrix for FAO [20]. Increased metabolic flexibility is necessary for augmentation of FAO when energy requirements increase, and failure to meet demands disturbs the relative distribution of acylcarnitines, thus reflecting mitochondrial metabolic dysfunction [19, 21, 22]. Defects in FAO limit ATP production when stressors such as infection, starvation, or oxidative stress increase metabolic demands [23–25]. Prior studies have shown that elevated plasma acetylcarnitine is associated with increased mortality in a sepsis cohort [26], while reduced carnitine transport has been implicated in ARDS and in a murine model of ventilator-induced lung injury [27].

While FAO defects may be associated with acute lung injury (ALI) and have prognostic implications in critical illness, acylcarnitine profiles in ARDS and their relationship to inflammatory biomarkers within the paradigm of known host-response subphenotypes is unclear. Furthermore, less is known about metabolites associated with outcomes in critically ill patients with acute respiratory failure independent of etiology. Such data would provide a framework for additional phenotyping to identify patients that may benefit from targeted therapies that replete energy stores during critical illness. The goal of this study is to determine whether serum acylcarnitine profiles differ between controls and ARDS subphenotypes, and if they are associated with plasma biomarkers and clinical outcomes in acute respiratory failure.

## Methods

### Clinical cohort: the Acute Lung Injury Registry at the University of Pittsburgh Medical Center

From October 2011 to January 2019, we prospectively enrolled adult patients admitted to intensive care units at UPMC Presbyterian-Montefiore Hospital with acute respiratory failure requiring intubation and mechanical ventilation. Exclusion criteria included inability to obtain informed consent. Over 90% of plasma and serum samples included in this study were collected within 72 h of intubation. We collected baseline demographics, comorbidities, mechanical ventilation parameters, laboratory variables, and calculated lung injury prediction scores (LIPS). All plasma and serum samples were processed using standard tube collection methods and were processed and stored at -80 degrees within 6 h of collection. All serum samples underwent no more than one freeze–thaw cycle prior to analysis and were batch processed for targeted metabolomics analysis. Modified sequential organ failure assessment (SOFA) scores were calculated at study enrollment that excluded Glasgow Coma Scale scores since patients were intubated and sedated as previously reported [9]. Patients within the cohort are classified into groups of ARDS, at-risk for ARDS, or not at-risk for ARDS (airway controls) by a consensus of at least 3 board-certified pulmonologists according to the Berlin definition. Plasma biomarkers analyzed included soluble tumor necrosis factor receptor 1 (TNFR1), bicarbonate, interleukin-6 (IL-6), interleukin-8 (IL-8), soluble receptor of advanced glycation end-products (RAGE), procalcitonin, and angiopoietin-2 [9, 28]. Patients intubated for airway protection without evidence of clinical ARDS were designated as airway controls and included in the study. Only patients meeting the Berlin definition of ARDS, not at-risk for ARDS, were included and were designated as Class 1 and Class 2 using predicted probabilities for subphenotypic classification by a parsimonious, logistic regression model utilizing plasma levels of TNFR1, IL-8, and bicarbonate. This model was derived from variables previously used in the latent class analysis (LCA) model in a randomized controlled trial of ARDS patients and independently validated for prognostic value in our patient population with and at-risk for ARDS [9, 28, 29]. These patients were stratified by TNFR1 levels. Class 1 and Class 2 associated with the highest and lowest quartiles of TNFR1 levels, respectively, were selected for inclusion (N = 50 per group). Airway controls (N = 50) were included as a comparator group, with 82% designated as Class 1 by the parsimonious model [28]. Outcomes included vasopressor use within seven days of enrollment and 30-day mortality across the cohort with acute respiratory failure inclusive of Class 1 ARDS, Class 2 ARDS, and airway controls.

### Targeted metabolomics analysis

For initial screening, targeted profiling analysis of serum was conducted for 25 amino acids along with carnitine and acetylcarnitine. Sample preparation, extraction, and initial targeted analysis are included in Additional file 1: Material and Table S1. Based on the changes in acetylcarnitine in Class 2 patients after profiling 150 patients, acylcarnitines were further analyzed by liquid chromatography high resolution mass spectrometry (LC-HRMS) in 129 patients based on sufficient sample available for these analyses. Samples were injected via a Thermo Vanquish UHPLC and separated over a reversed phase Waters Aquity BEH C18 column (2.1 × 100 mm, 1.7 μm particle size) maintained at 55 °C. The 20 min LC gradient used a flow rate of 200 µL/min and the mobile phase consisted of solvent A (water/0.1% FA) and solvent B (acetonitrile/0.1% FA). The gradient was the following: 0–0.1 min 2%B, increase to 20%B over 6 min, with a further increase to 95%B over 9 min, holding at 95%B for three minutes, and equilibrating at initial conditions of 2%B for 7 min. The Thermo ID-X tribrid mass spectrometer was operated in positive ion mode, scanning in ddMS^2^ mode (2 μscans) from 70 to 800 m*/z* at 120,000 resolution with an AGC target of 2e5 for full scan, 2e4 for MS^2^ scans using HCD fragmentation at stepped 15, 35, and 50 collision energies. Source ionization spray voltage was set to 3.0 kV. Source gas parameters were 35 sheath gas, 12 auxiliary gas at 320 °C, and 8 sweep gas. Calibration was performed prior to analysis using the PierceTM FlexMix Ion Calibration Solutions (Thermo Fisher Scientific). Integrated peak areas were then extracted manually using Quan Browser (Thermo Fisher Xcalibur ver. 2.7). Relative amounts of acylcarnitines are reported as the peak area ratio of the analyte to their corresponding deuterated internal standard for serum samples, and levels for metabolites analyzed were detectable in all samples.

### Statistics

For clinical and demographic data, the pairwise Mann–Whitney U test was performed for pairwise comparisons among controls, Class 1, and Class 2 groups. Relative quantities using integrated peak area ratios of metabolite levels normalized to internal isotope-labeled controls were analyzed across all groups (airway controls, Class 1, and Class 2) using the Kruskal–Wallis test with post-hoc Dunn’s multiple comparisons test. Pairwise Spearman correlation coefficients were calculated for plasma biomarkers and serum metabolites using Stata (version 17, StataCorp LLC). When comparing the free carnitine/palmitoylcarnitine + oleoylcarnitine ratio between controls and all ARDS patients within the cohort studied, the Mann–Whitney U test was used for pairwise comparison of the two groups using Prism 9 Graph Pad software (Version 9.3.1). Quantile g-computation (QGC) modeling using the R package “qgcomp” was performed [30–32] for ARDS subphenotypes. A detailed description of QGC is included in Additional file 1: Material with calculations.

## Results

### Demographics and clinical characteristics of defined ARDS subphenotypes

For the study design, patients were initially classified as airway controls, Class 1, and Class 2 ARDS subphenotypes using the parsimonious model within our cohort with N = 50 per group [8, 9, 29]. Class 1 and Class 2 ARDS patients selected represented the lowest and highest quartile of TNFR1 biomarker levels, respectively (Additional file 2: Figure S1). Increased BMI was observed in the Class 1 subphenotype relative to all other groups (*P* < 0.01 for Class 2, *P* < 0.001 for controls, Table 1). Preexisting diagnoses including diabetes, chronic obstructive pulmonary disease (COPD), congestive heart failure, renal failure, and immunosuppression were similar among groups with the exception of alcohol use which was higher in airway controls relative to Class 1 (*P* = 0.031). Underlying causes of ARDS, most commonly sepsis and pneumonia, and nadir PaO2:FiO2 ratios during hospitalization were similar in Class 1 and Class 2 (*P* = 0.68 and *P* = 0.14, respectively). Class 2 patients had higher lung injury prediction score (LIPS), sequential organ failure assessment (SOFA) scores, and percent of individuals with acute kidney injury (Table 2). Clinical parameters including respiratory mechanics (peak end expiratory pressure and plateau pressure), ventilator-free days, vasopressor use, and 30-day mortality were similar between ARDS groups within the nested case–control cohort. For plasma biomarkers, bicarbonate levels were increased in Class 1 patients whereas IL-6, procalcitonin, IL-8, RAGE, TNFR1, and angiopoietin-2 levels were all increased in the Class 2 group as expected (Additional file 2: Figure S1) [9, 10].

**Table 1:** Demographic data for Acute Lung Injury Registry cohort

Variable	Class 1 ARDS (N = 50)	Class 2 ARDS (N = 50)	Airway controls (N = 50)	*P*-value Class 1 vs. Class 2	*P*-value Class 1 vs. Controls	*P*-value Class 2 vs. Controls
Age, median [IQR], years	56.0 [45.5, 64.4]	56.5 [40.8, 65.5]	54.0 [43.7, 63.3]	0.83	0.74	0.76
Males, N (%)	28 (56.0)	25 (50.0)	28 (56.0)	0.69	> 0.99	0.69
BMI, median [IQR]	33.5 [28.3, 37.7]	28.6 [24.8, 32.2]	26.7 [23.9, 29.6]	0.0035	0.0005	0.25
Caucasian, N (%)	48 (96.0)	47 (94.0)	46 (92.0)	> 0.99	0.68	> 0.99
Non-Hispanic, N (%)	49 (98.0)	50 (100.0)	50 (100.0)	> 0.99	> 0.99	> 0.99
Time from intubation to sample collection, median [IQR], days	1.5 [1, 3]	1 [1, 2]	1 [1, 2]	0.29	0.0045	0.025
History of chronic disease						
Diabetes, N (%)	15 (30.0)	16 (32.0)	14 (28.0)	> 0.99	> 0.99	0.83
COPD, N (%)	7 (14.0)	11 (22.0)	6 (12.0)	0.44	> 0.99	0.29
Congestive heart failure, N (%)	4 (8.0)	3 (6.0)	3 (6.0)	> 0.99	> 0.99	> 0.99
Chronic lung disease, N (%)	6 (12.0)	5 (10.0)	8 (16.0)	> 0.99	0.77	0.55
Chronic renal failure, N (%)	4 (8.0)	11 (22.0)	6 (12.0)	0.09	0.74	0.29
Immunosuppression, N (%)	7 (14.0)	14 (28.0)	10 (20.0)	0.14	0.60	0.48
Alcohol Use, N (%)	4 (8.0)	7 (14.0)	13 (26.0)	0.53	0.031	0.21
Risk factors for ARDS						
Pneumonia, N (%)	34 (68.0)	31 (62.0)	0 (0.0)	0.68	< 0.001	< 0.001
Sepsis, N (%)	14 (28.0)	13 (26.0)	0 (0.0)	> 0.99	< 0.001	< 0.001
Pancreatitis, N (%)	2 (4.0)	2 (4.0)	0 (0.0)	> 0.99	0.50	0.50
Aspiration, N (%)	5 (10.0)	12 (24.0)	0 (0.0)	0.11	0.056	< 0.001
LIPS score, median [IQR]	6.5 [4.5, 8.5]	7.3 [4.8, 9.8]	3 (1.0, 5.0)	0.016	< 0.0001	< 0.0001
Severity of illness						
SOFA score, median [IQR]	7 [5, 9]	9 [7, 10]	5 [4, 7]	0.0138	0.0003	< 0.0001
Worst PaO2:FiO2 ratio, median [IQR], mm Hg	110.5 [84.0, 175.0]	137.5 [93.0, 202.0]	205.0 [164.0, 273.0]	0.14	< 0.0001	0.0001

Pairwise comparisons between groups were analyzed by the Mann–Whitney U test with *P*-values shown for each comparison. *IQR* interquartile range, *BMI* body mass index, *COPD* chronic obstructive pulmonary disease, *LIPS* Lung Injury Prediction Score, *SOFA* Sequential Organ Failure Assessment Score

**Table 2:** Laboratory and clinical data for Acute Lung Injury Registry cohort

Variable	Class 1 ARDS (N = 50)	Class 2 ARDS (N = 50)	Airway controls (N = 50)	*P*-value Class 1 vs. Class 2	*P*-value Class 1 vs. Controls	*P*-value class 2 vs. Controls
Laboratory parameters						
WBC, median [IQR], × 10^9^ per liter	12.8 [9.8, 17.2]	14.6 [10.4, 19.1]	10.8 [8.4, 15.3]	0.25	0.23	0.025
Platelets, median [IQR], × 10^9^ per liter	192.0 [139.0, 249.0]	149.5 [70.0, 244.0]	177.0 [113.0, 238.0]	0.14	0.46	0.33
Hgb, median [IQR], g/dL	9.9 [8.9, 10.9]	10.1 [8.2, 10.7]	11.8 [10.2, 13.5]	0.49	< 0.0001	< 0.0001
BUN, median [IQR], mg/dL	28.5 [15.0, 45.0]	41.0 [23.0, 54.0]	18.0 [10.0, 26.0]	0.041	0.0009	< 0.0001
Creatinine, median [IQR], mg/dL	1.0 [0.7, 2.0]	2.6 [1.4, 3.6]	0.9 [0.6, 1.2]	< 0.0001	0.12	< 0.0001
Glucose, median [IQR], mg/dL	137.0 [106.5, 170.0]	137.0 [118.0, 154.0]	134.0 [107.0, 173.0]	0.71	0.61	0.90
Mechanical ventilation parameters						
PEEP, median [IQR], cm H2O	10.0 [8.0, 12.0]	8.0 [5.0, 12.0]	5.0 [5.0, 5.0]	0.28	< 0.0001	< 0.0001
Plateau Pressure, median [IQR], cm H2O	26.0 [22.0, 30.0]	25.0 [21.0, 31.0]	16.0 [13.0, 18.0]	0.60	< 0.0001	< 0.0001
Outcomes						
Acute Kidney Injury, N (%)	20 (40.0)	36 (72.0)	11 (22.0)	0.002	0.083	< 0.001
Duration of intubation, median [IQR], days	11.0 [7.0, 17.0]	7.0 [5.0, 12.0]	4.0 [2.0, 7.0]	0.014	< 0.0001	< 0.0001
ICU LOS, median [IQR], days	14.0 [9.0, 21.0]	10.5 [7.0, 18.0]	5.0 [4.0, 10.0]	0.069	< 0.0001	< 0.0001
VFD, median [IQR], days	11.0 [0.0, 20.0]	1.0 [0.0, 22.0]	24.0 [19.0, 26.0]	0.96	< 0.0001	< 0.0001
Vasopressor Use, N (%)	33 (66.0)	37 (74.0)	8 (16.0)	0.38	< 0.0001	< 0.0001
30 Day mortality, N (%)	15 (30.0)	17 (34.0)	7 (14.0)	0.83	0.090	0.034
90 Day mortality, N (%)	18 (36.0)	18 (36.0)	8 (16.0)	> 0.99	0.039	0.039

Pairwise comparisons between groups were analyzed by the Mann–Whitney U test with *P*-values shown for each comparison. Abbreviations: *WBC* white blood cell count, *Hgb* hemoglobin, *IQR* interquartile range, *PEEP* positive end-expiratory pressure, *LOS* length of stay, *VFD* ventilator-free days

### Acylcarnitine profiles in clinical subphenotypes of ARDS

**Fig. 1:**
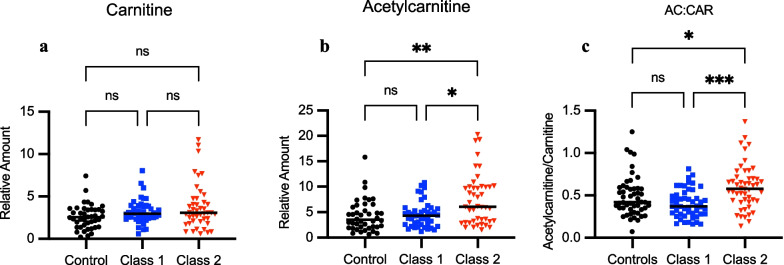
Carnitine and acetylcarnitine levels for controls, Class 1, and Class 2 ARDS groups. Relative amounts (peak area ratio of analyte/internal standard) of metabolites shown were determined using stable isotope dilution liquid chromatography high resolution mass spectrometry (LC-HRMS). Controls are shown as black circles (n = 50), Class 1 as blue squares (n = 50), and Class 2 (n = 50) as red triangles with black bars representing medians. Relative carnitine levels are shown in (**a**) and (**b**) relative acetylcarnitine levels. **c** Acetylcarnitine/carnitine ratios are presented for airway controls, Class 1, and Class 2 groups. Group comparisons were performed using the Kruskal–Wallis test. Asterisks indicate *P* < 0.05 (*), *P* < 0.01 (**), *P* < 0.001 (***),  and ns (not significant)

**Fig. 2:**
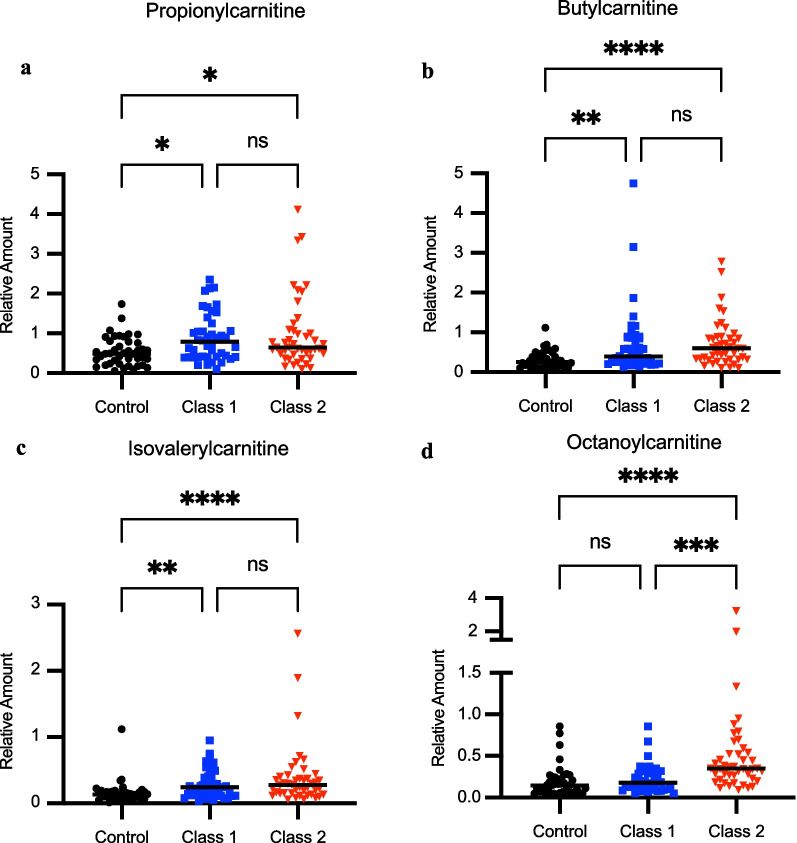
Acylcarnitine profiles distinguish Class 1 and Class 2 ARDS groups. Serum levels of short and medium-chain acylcarnitines are shown. Relative amounts (peak area ratio of analyte/internal standard) of metabolites shown were determined using stable isotope dilution liquid chromatography-high resolution mass spectrometry (LC-HRMS). Controls (n = 42) are shown as black circles, Class 1 (n = 44) as blue squares, and Class 2 (n = 43) as red triangles with black bars representing medians. Panels show relative levels for (**a**) propionylcarnitine, (**b**) butyrylcarnitine, (**c**) isovalerylcarnitine, and (**d**) octanoylcarnitine. Group comparisons were performed using the Kruskal–Wallis test. Asterisks indicate *P* < 0.05 (*), *P* < 0.01 (**), *P* < 0.001 (***), *P* < 0.0001 (****), and ns (not significant)

The targeted profiling analysis performed on the serum samples showed a significant increase in acetylcarnitine for Class 2 ARDS in the cohort. To further characterize changes in acylcarnitines, a targeted LC-HRMS analysis was conducted for 129 patients. Free carnitine represents approximately 83% of the carnitine pool while 17% is comprised of acylcarnitines, with acetylcarnitine representing 75% of this portion [19, 20, 33, 34]. Carnitine levels were similar between all groups (Table 3, Fig. 1a). However, Class 2 patients showed 73% and 41% increase in median acetylcarnitine levels relative to controls and Class 1 ARDS patients, respectively (Table 3, Fig. 1b). A serum acetylcarnitine/carnitine ratio of greater than 0.4 generally reflects mitochondrial metabolic dysfunction [19, 20]. In Class 2 ARDS, the median ratio was 0.58 compared to ratios of 0.37 for Class 1 and 0.42 for airway controls (Table 3, Fig. 1c). We also observed negative correlation with bicarbonate (ρ = − 0.38, *P* < 0.0001) and positive correlations with TNFR1 (ρ = 0.31, *P* = 0.0001) and RAGE levels (ρ = 0.28, *P* = 0.0005) (Additional file 2: Figure S2). We then determined if there were differences in other acylcarnitine species between groups through targeted profiling. Data showed significant increases of several short chain acylcarnitines found in both classes of ARDS relative to airway controls. For example, propionylcarnitine (C3) was increased in both ARDS classes relative to airway controls in addition to butyrylcarnitine (C4) and isovalerylcarnitine (C5) (Table 4, Fig. 2a–c). However, octanoylcarnitine, a medium-chain acylcarnitine, was the most significantly increased acylcarnitine measured in the Class 2 group with levels 2-fold higher than Class 1 and airway controls (Table 4, Fig. 2d**)**.

**Table 3:** Relative acetylcarnitine and carnitine levels across classes in patients with acute respiratory failure

Metabolite	Airway controls (N = 50)	Class 1 ARDS (N = 50)	Class 2 ARDS (N = 50)	Fold change Class 1 vs. controls	*P*-value	Fold change Class 2 vs. controls	*P*-value	Fold Change Class 2 vs Class 1	*P*-value
Carnitine (C0) [Median, IQR]	2.54 [1.39, 3.46]	2.94 [2.44, 3.88]	3.07 [2.04, 4.63]	1.16	0.21	1.21	0.11	1.04	1.00
Acetylcarnitine (C2) [Median, IQR]	3.51 [2.05, 6.09]	4.30 [2.57, 5.59]	6.08 [3.19, 9.99]	1.23	1.00	1.73	0.0016	1.41	0.016
C2:C0 [Median, IQR]	0.42 [0.33, 0.59]	0.37 [0.28, 0.51]	0.58 [0.43, 0.70]	0.88	0.30	1.38	0.043	1.56	0.0001

Median values are shown for relative amounts (peak area ratio of analyte/internal standard) of metabolites as determined using stable isotope dilution liquid chromatography high resolution mass spectrometry (LC-HRMS). Interquartile range (IQR) is shown for each metabolite with fold-change and *P*-values among airway controls, Class 1 ARDS, and Class 2 ARDS patients. Relative amounts were compared using the Kruskal–Wallis test with Dunn’s multiple comparisons test. The median ratio of acetylcarnitine to carnitine (C2:C0) is also shown with IQR included. Groups were compared using the Kruskal–Wallis test with *P*-values as shown

**Table 4:** Relative levels acylcarnitines across classes within Acute Lung Injury Registry cohort

Metabolite	Airway controls (N = 42)	Class 1 ARDS (N = 44)	Class 2 ARDS (N = 44)	Fold change Class 1 vs. controls	*P*-value	Fold change Class 2 vs. controls	*P*-value	Fold change Class 2 vs Class 1	*P*-value
Propionylcarnitine [Median, IQR]	0.49 [0.24, 0.80]	0.79 [0.41, 1.21]	0.65 [0.48, 1.08]	1.62	0.14	1.32	0.027	0.82	1.00
Butyrylcarnitine [Median, IQR]	0.26 [0.15, 0.45]	0.40 [0.26, 0.87]	0.60 [0.32, 0.88]	1.52	0.0012	2.29	< 0.0001	1.51	1.00
Isovalerylcarnitine [Median, IQR]	0.14 [0.078, 0.18]	0.25 [0.12, 0.40]	0.28 [0.15, 0.39]	1.81	0.0017	2.04	< 0.0001	1.13	0.96
Octanoylcarnitine [Median, IQR]	0.15 [0.059, 0.25]	0.18 [0.10, 0.32]	0.35 [0.22, 0.54]	1.20	0.29	2.34	< 0.0001	1.94	0.00040
Myristoylcarnitine[Median, IQR]	0.035 [0.023, 0.059]	0.028 [0.022,0.047]	0.033 [0.023,0.062]	0.82	0.49	0.95	> 0.9999	1.16	0.60

Median values are shown for relative amounts (peak area ratio of analyte/internal standard) of metabolites as determined using stable isotope dilution liquid chromatography high resolution mass spectrometry (LC-HRMS). Interquartile range (IQR) is shown for each metabolite with fold-change and *P*-values among airway controls, Class 1 ARDS, and Class 2 ARDS patients

### Increased octanoylcarnitine is associated with the class 2 ARDS subphenotype

**Fig. 3:**
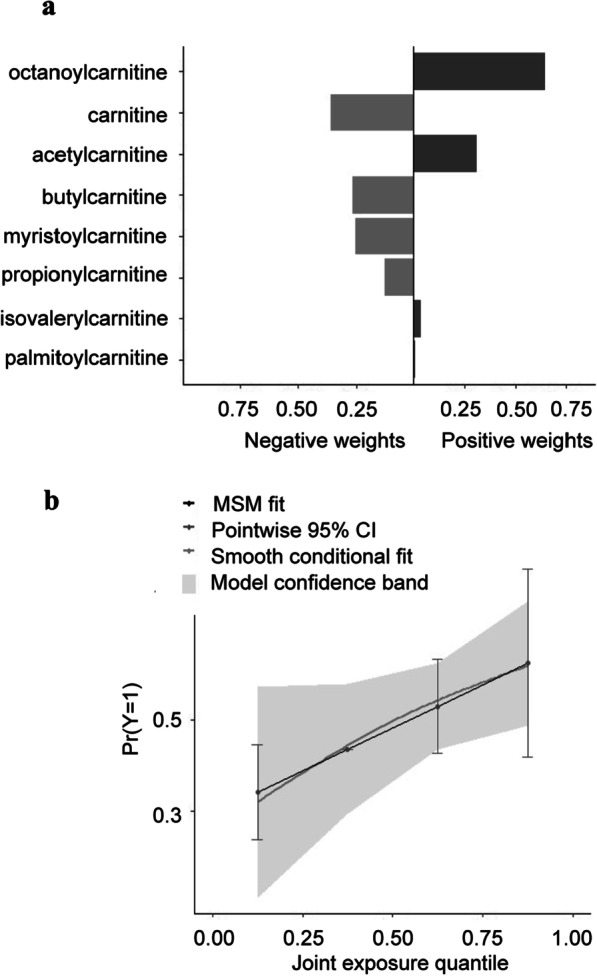
Increased levels of octanoylcarnitine are most closely associated with Class 2 ARDS. Quantile-based g-computation analysis was performed to determine which acylcarnitines were most likely to distinguish between Class 1 and Class 2 ARDS while correcting for age, sex, and creatinine as a measure of renal function. **a** Acylcarnitines were ranked by weights for Class 2 relative to Class 1 ARDS, (**) indicating *P* = 0.0014. **b** Graph shows the probability Pr(Y = 1) of the Class 2 phenotype plotted against joint exposure quantile with 95% confidence interval shown by the gray line. The black solid line shows the marginal structural model (MSM) and smooth conditional fit by the gray solid line

Within our targeted acylcarnitine analysis, we sought to determine which acylcarnitines profiled were driving the differences between the two ARDS subphenotypes. The quantile-based g-computational method (QGC) has been used with increasing frequency to better understand the role of exposures and mixture effects on outcome [30–32]. Given the complexity of exposures and suspected mixture effects when examining the metabolome within the control and ARDS populations, we used QGC with bootstrapping to examine joint exposures for individual serum acylcarnitine levels [35, 36]. Renal tubular reabsorption of acylcarnitines in normal renal function is highly efficient [19, 37]. When adjusting the model for age, sex, and creatinine as a measure of renal function, octanoylcarnitine was most closely associated with the Class 2 subphenotype (*P* = 0.004), while acetylcarnitine was not statistically significant (*P* = 0.16) (Fig. 3a, b**)**. Thus, octanoylcarnitine is a potential serum biomarker for the Class 2 ARDS subphenotype. The mixture effect of each metabolite on the risk of Class 2 (adjusted for other metabolites) was not statistically significant (P = 0.07) by this analysis.

### Acylcarnitine levels positively correlate with inflammatory biomarkers and creatinine

**Fig. 4:**
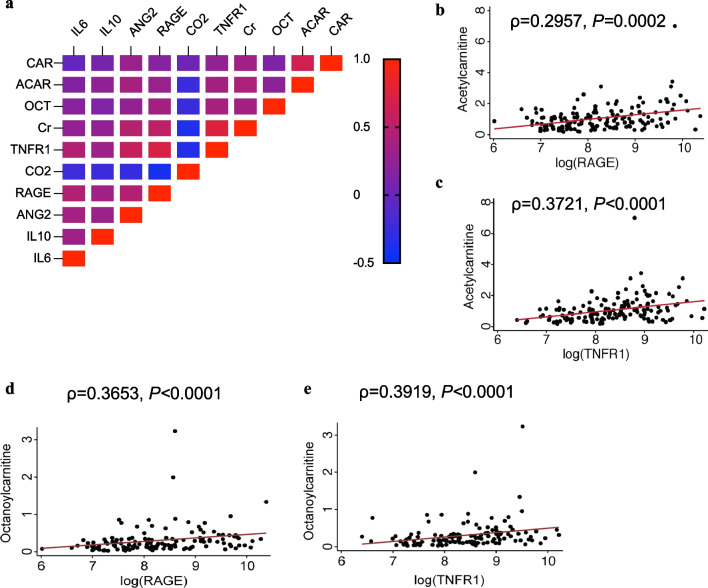
Acetylcarnitine and octanoylcarnitine levels positively correlate with inflammatory biomarkers. **a** Correlation matrix is shown for log (concentration) of biomarkers and relative amounts of carnitine (CAR), acetylcarnitine (ACAR), and octanoylcarnitine (OCT) with log concentrations of serum biomarkers creatinine (Cr), TNFR1, bicarbonate (CO2), RAGE, angiopoietin-2 (ANG2), IL-10, and IL-6 within the cohort. Pairwise correlations are shown for acetylcarnitine and (**b**) RAGE and (**c**) TNFR1 with Spearman’s coefficient (ρ) and *P* values. Pairwise correlations are shown for octanoylcarnitine and log concentrations of (**d**) RAGE and (**e**) TNFR1 with Spearman’s coefficient (ρ) and *P*-values

**Fig. 5:**
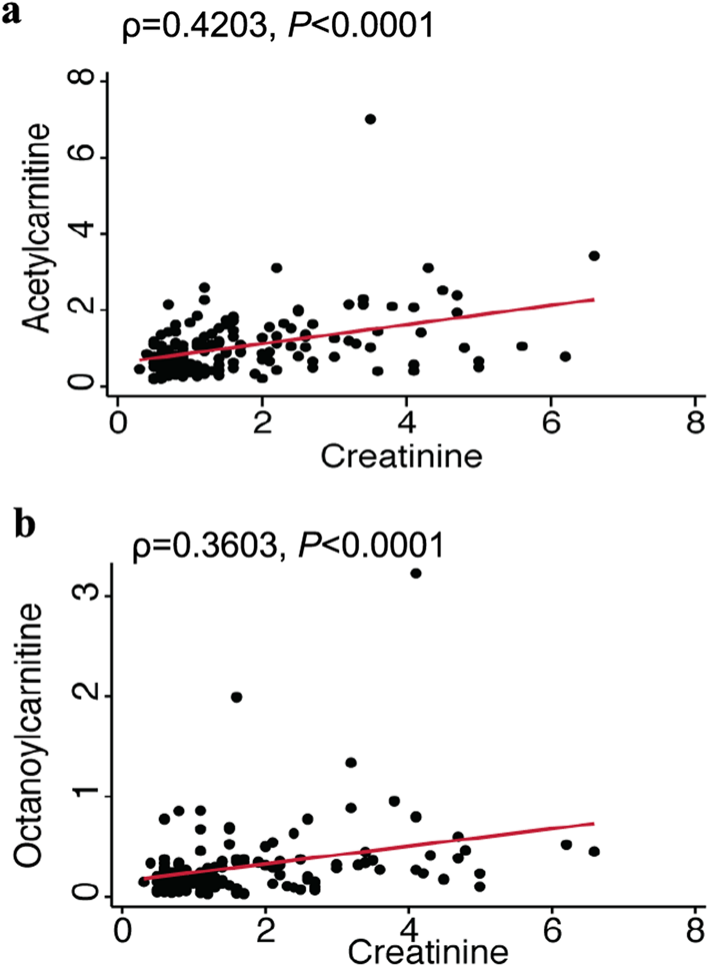
Acetylcarnitine and octanoylcarnitine levels positively correlate with renal dysfunction. Pairwise correlations are shown for creatinine and (**a**) acetylcarnitine and (**b**) octanoylcarnitine. Spearman’s coefficient (ρ) and *P*-values are shown

Given the findings that serum acetylcarnitine and octanoylcarnitine levels are elevated in the Class 2 subphenotype, we sought to determine if serum biomarkers previously incorporated into multiple models for ARDS classification correlated with these metabolites (Fig. 4a). Both octanoylcarnitine and acetylcarnitine positively correlated with RAGE and TNFR1 levels (Fig. 4b–e). Levels also positively correlated with creatinine (ρ = 0.4203, *P* < 0.0001 for acetylcarnitine and ρ = 0.3603, *P* < 0.0001 for octanoylcarnitine) (Fig. 5a, b). A recent study showed that plasma free carnitine/palmitoylcarnitine + oleoylcarnitine ratio (C0/C16 + C18), a surrogate measure of carnitine palmitoyltransferase (CPT1) activity, is reduced in ARDS patients relative to controls. These findings did not incorporate host-response subphenotype classification but showed that patients with an increased C0/C16 + C18 ratio had elevated plasma RIPK3 levels [27]. We examined our own cohort for evidence of acquired CPT1 deficiency associated with specific ARDS classes (Additional file 2: Figure S3). Our data confirmed an overall increase in C0/C16 + C18 in ARDS patients relative to airway controls consistent with the previously published study, but there was no statistically significant difference between Class 1 and Class 2 subphenotypes (Additional file 2: Figure S3) [27].

### Targeted profiling of serum revealed changes in 3-methylhistidine in the Class 2 phenotype

**Fig. 6.**
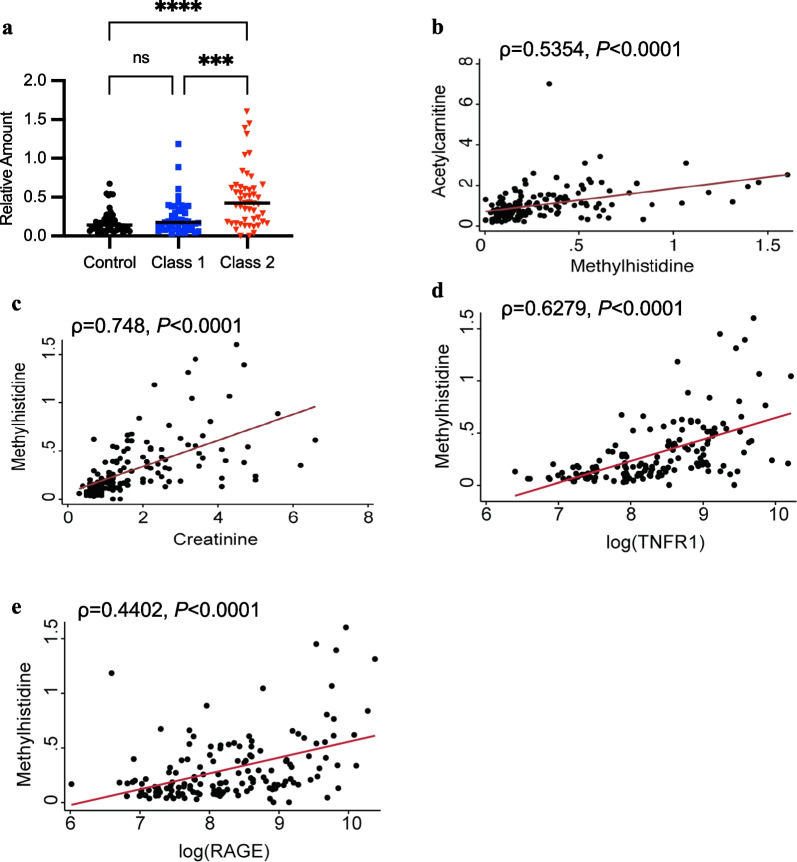
3-Methylhistidine is increased in Class 2 ARDS patients. **a** Relative amounts (peak area ratio of analyte/internal standard) of 3-methylhistidine shown were determined using stable isotope labeled liquid chromatography high resolution mass spectrometry. Controls are shown as black circles, Class 1 as blue squares, and Class 2 as red triangles with black bars representing medians. Group comparisons were performed using the Kruskal–Wallis test. Asterisks indicate *P* < 0.001 (***), *P* < 0.0001(****), and ns (not significant). **b–e** show Spearman correlation coefficients (ρ) for 3-methylhistidine with (**b**) acetylcarnitine, (**c**) creatinine, and log concentrations of (**d**) TNFR1 and (**e**) RAGE

During critical illness, inefficient energy production and utilization contributes to increased muscle catabolism [38, 39]. Increased serum concentrations and urinary excretion of 3-methylhistidine (3-MH) are associated with skeletal muscle breakdown and renal dysfunction [40–43]. Given the importance of fatty acid oxidation and carnitine metabolism to meet energy needs under stress in skeletal muscle, we were intrigued to find 3-MH, a biomarker of muscle catabolism, to be elevated in Class 2 ARDS. Within our cohort, median 3-MH levels were increased more than twofold in Class 2 ARDS relative to Class 1 and 2.5-fold higher than controls (Fig. 6a). We also observed positive correlations with renal dysfunction (increasing creatinine), acetylcarnitine levels, RAGE, and TNFR1 across all groups (Fig. 6b–e).

### Increased serum levels of 3-methylhistidine and octanoylcarnitine in acute respiratory failure are associated with worse clinical outcomes

**Fig. 7:**
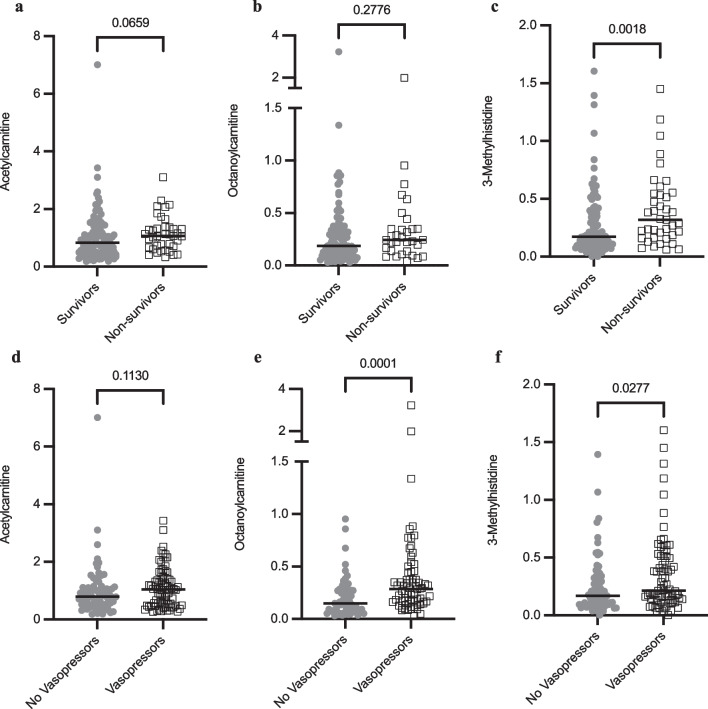
Increased 3-methylhistidine and octanoylcarnitine levels are associated with 30-day mortality and vasopressor use in patients with acute respiratory failure. The cohort includes airway controls, Class 1, and Class 2 ARDS patients and was stratified by 30-day survival (**a–c**) and vasopressor use within 7 days of study enrollment (**d–f**). **a** Relative acetylcarnitine levels are shown for survivors (N = 111) and non-survivors (N = 39). **b** Relative octanoylcarnitine levels are shown for survivors (N = 98) and non-survivors (N = 32). **c** Relative 3-methylhistidine levels are shown for survivors (N = 111) and non-survivors (N = 39). **d** Relative acetylcarnitine levels are shown for patients not on vasopressors (N = 72) and those requiring vasopressors (N = 78). **e** Relative octanoylcarnitine levels are shown for patients not on vasopressors (N = 60) and those requiring vasopressors (N = 70). **f** Relative 3-methylhistidine levels are shown for patients not on vasopressors (N = 72) and those requiring vasopressors (N = 78). Statistical analysis was performed using the Mann–Whitney U test with *P*-values shown

We next asked whether 3-MH, acetylcarnitine, and octanoylcarnitine levels were increased in patients who required vasopressor use within seven days from the time of enrollment and in patients who died within 30 days of initiation of mechanical ventilation (Table 5). Acetylcarnitine levels did not differ among survivors, non-survivors, or in patients with or without vasopressor support (Fig. 7a, d). While octanoylcarnitine levels were similar in survivors and non-survivors, levels were increased twofold in patients who required vasopressors compared to those who did not (Fig. 7b, e). Median levels of 3-MH were also increased twofold in non-survivors compared to survivors (Fig. 7c). Patients requiring vasopressors showed a 20% increase in 3-MH compared to those that did not which was statistically significant (*P* = 0.027) (Fig. 7f).

**Table 5:** Relative metabolite levels in survivors, non-survivors, and vasopressor-dependent patients with acute respiratory failure within the cohort

	Survivors (N = 111)	Non-survivors (N = 39)	*P*-value	No Vasopressors (N = 72)	Vasopressors (N = 78)	*P*-value
Acetylcarnitine [Median, IQR]	0.83 [0.49, 1.31]	1.07 [0.67, 1.37]	0.066	0.79 [0.51, 1.13]	1.04 [0.52, 1.44]	0.11

	Survivors (N = 98)	Non-survivors (N = 32)	*P*-value	No Vasopressors (N = 60)	Vasopressors (N = 70)	*P*-value
Octanoylcarnitine [Median, IQR]	0.19 [0.11, 0.35]	0.24 [0.13, 0.35]	0.28	0.15 [0.075, 0.28]	0.29 [0.16, 0.44]	**0**.**0001**

	Survivors (N = 111)	Non-survivors (N = 39)	*P*-value	No Vasopressors (N = 72)	Vasopressors (N = 78)	*P*-value
3-Methylhistidine [Median, IQR]	0.17 [0.095, 0.36]	0.32 [0.16, 0.55]	**0**.**0018**	0.17 [0.095, 0.33]	0.21 [0.13, 0.51]	**0**.**028**

Relative acetylcarnitine, octanoylcarnitine, and 3-methylhistidine levels (peak area ratio of analyte/internal standard) are shown among survivors, non-survivors, and patients who did or did not require vasopressors within seven days of study enrollment. Pairwise comparisons between groups were analyzed by the Mann–Whitney U test with *P*-values shown for each comparison

## Discussion

Elevated plasma acetylcarnitine has been observed in sepsis and linked to increased mortality [26]. Despite similar proportions of patients with sepsis in both ARDS classes within our cohort, we observed increased acetylcarnitine and acetylcarnitine/carnitine ratios in Class 2, suggesting disproportionate impact of metabolic dysregulation within this subphenotype. Our study also shows increased short-chain acylcarnitines in both ARDS groups relative to airway controls. Elevated serum octanoylcarnitine suggests that medium-chain fatty acid utilization is reduced in Class 2 and associated with vasopressor use in patients with acute respiratory failure across the cohort. Altered serum acylcarnitine profiles in this study provide evidence for fatty acid oxidation defects and mitochondrial metabolic dysfunction as a characteristic of the Class 2 hyperinflammatory subphenotype. These findings are consistent with a prior study that examined this subphenotype within a single center cohort of ARDS patients with concurrent sepsis [18]. In this study by Alipanah-Lechner and colleagues, untargeted metabolomics profiling of a sepsis cohort with ARDS revealed lower levels of lipids and higher levels of glycolytic metabolites consistent with dysregulated lipid metabolism in Class 2 relative to Class 1 [18]. Our study supports these findings and suggests that mitochondrial metabolic dysfunction may have prognostic significance in critical illness associated with acute respiratory failure independent of the etiology.

Acylcarnitines are measured in the clinical setting, usually for diagnosis of inborn errors of metabolism in the pediatric population [20, 22]. Elevated serum levels of octanoylcarnitine is diagnostic of medium-chain acyl-coenzyme A dehydrogenase deficiency (MCAD) which presents as lethargy, hypoglycemia, and gastrointestinal symptoms in the neonatal period or during periods of physiologic stress [44, 45]. Decreased CPTI and carnitine/acylcarnitine translocase (CACT) activity can also increase octanoylcarnitine [22, 46]. Statins can increase CPT1 activity in vitro and in vivo, and may augment carnitine transport through pleotropic mechanisms [47–50]. An intriguing result from a secondary analysis showed that statin therapy was associated with reduced mortality and increased ventilator-free days in Class 2, but not Class 1 ARDS patients [10]. These findings are of particular interest since statins have been shown to increase fatty acid oxidation in multiple studies [48, 51, 52]. Acylcarnitines also reflect increased oxidative stress and insulin resistance in skeletal muscle in vitro, supporting their use as reliable markers of increased oxidative stress in systemic illness [34]. FAO defects and accumulation of long chain acylcarnitines disrupt surfactant function and exacerbate acute lung injury [33, 53, 54]. Long-chain acylcarnitines are difficult to measure in serum as they are present at low levels [19]. Since serum and plasma acyl-CoA moieties are exceedingly difficult to measure by analytical methods, acylcarnitines continue to be the best surrogate measure of FAO [19, 20]. Our data illustrate increased short and medium-chain acylcarnitines in both ARDS classes, whereas long-chain acylcarnitines such as oleoylcarnitine (C16) and palmitoylcarnitine (C18) were not significantly different among airway controls and ARDS classes. Collectively, these data raise questions about how unfavorable cellular metabolism may increase susceptibility to tissue injury and multi-organ dysfunction in critical illness and contribute to the “reactive” phenotype of the Class 2 group.

3-MH was increased in Class 2 ARDS despite no significant differences in the underlying etiologies of ARDS within the cohort. In sepsis, inflammatory responses triggered by endotoxin induce skeletal muscle catabolism, with increased 3-MH levels reflecting skeletal muscle atrophy and fiber loss during prolonged critical illness [40, 41]. In severe COPD, serum 3-MH was increased in patients with cachexia [39, 55, 56]. Our observation that serum 3-MH is increased in Class 2 may both reflect a catabolic state and decreased renal clearance since acute kidney injury is strongly associated with the Class 2 subphenotype [7, 8]. Using targeted metabolomics, our data suggest that Class 2 represents a hypercatabolic state relative to Class 1, with increased 3-MH reflecting skeletal muscle protein breakdown during acute critical illness.

While we utilized serum samples to identify metabolites associated with ARDS subphenotypes, it is unclear how differences in profiles across tissue compartments will predict outcomes or responses to therapies. Serum acylcarnitines including those profiled in this study correlate well with plasma levels [57]. Further correlation between serum and bronchoalveolar lavage fluid or exhaled respiratory samples targeting specific groups of metabolites could provide complementary tissue-targeted data [14, 58, 59]. For example, in a small cohort of patients with ARDS from influenza A infection, 3-MH was reduced in the bronchoalveolar lavage fluid (BALF) in contrast to our serum findings [60]. In addition, the prevalence of renal failure in the Class 2 group impacts several metabolites including acylcarnitines [43, 61]. Our QGC model highlighted octanoylcarnitine as a metabolic biomarker distinguishing ARDS classes independent of renal function without evidence of mixing effects from other acylcarnitines analyzed in this study. In an experimental model of ischemic acute kidney injury, lung, kidney, and plasma metabolomes were altered with marked deficiencies in fatty acid oxidation as the most significant change [62]. The mechanistic links between acute kidney injury and acute lung injury remain unclear, but growing evidence support the concept that metabolic derangements exacerbated by renal dysfunction may worsen tissue injury and systemic illness [63].

Selection and sample size are the limitations in our study and the likely factors contributing to similar outcomes for vasopressor use and 30-day mortality in selected Class 1 and Class 2 ARDS patients, findings that are in contrast to large multicenter studies or the entire cohort from our prior studies [3, 4, 7]. The patients included in this study had diverse etiologies of ARDS within a limited sample size for each class. TNFR1 was a major variable in patient selection across subgroups from the larger cohort and positively correlated with acetylcarnitine, octanoylcarnitine, and 3-MH levels in all patients with acute respiratory failure in the study. Prior studies have shown that the 2-class model of host-response subphenotypes is not only relevant in ARDS, but also in patients at-risk for ARDS and patients with acute respiratory failure [9, 28, 29, 64]. Even when utilizing predicted probabilities from externally developed parsimonious models using TNFR1, IL-8, and bicarbonate and not de-novo LCA for each cohort, this 2-class model is valid. Therefore, our data suggest that octanoylcarnitine and 3-MH may be surrogates of inflammation and fit into the framework of host-response subphenotypes in critically ill patients with acute respiratory failure irrespective of etiology [9, 28]. Obesity has been shown to be protective from sepsis-induced skeletal muscle catabolism [65]. We observed a trend for higher body mass index (BMI) in the Class 1 group, but this may also be related to sample size. Larger studies sufficiently powered for outcomes with external validation will be needed to determine generalizability of these findings.

## Conclusions

In summary, acylcarnitines are increased in ARDS with Class 2 “hyperinflammatory” patients showing metabolic profiles consistent with a catabolic phenotype and suppressed FAO. In particular, increased octanoylcarnitine is most closely associated with the Class 2 subphenotype and vasopressor use while increased 3-methylhistidine seemed to be associated with mortality across the cohort. Additional studies are warranted to determine if further subphenotyping within ARDS cohorts can identify populations of patients with greater FAO defects, and to assess generalizability of acylcarnitine profiling in ARDS patients. Targeting pathways to optimize mitochondrial function during acute illness may be feasible and beneficial in select patients. Our findings provide a framework for precision medicine in ARDS that incorporates metabolomics that may guide selection of targeted therapies in this diverse and challenging patient population.

## Supplementary Information

**Additional file 1.** Supplemental methods for targeted metabolomics and quantile g-computation modeling.

**Additional file 2.** Supplemental data including biomarker profiles and additional acylcarnitine analyses.

## Data Availability

All data generated and analyzed during this study are included in this published article and its supplementary information files.

## Abbreviations

FAOD: Fatty acid oxidation defects
ARDS: Acute respiratory distress syndrome
3-MH: 3-Methylhistidine
CACT: Carnitine/acylcarnitine translocase
CPT1: Carnitine palmitoyltransferase 1
