# Supplementary material for: Serum metabolomic signatures of fatty acid oxidation defects differentiate host-response subphenotypes of acute respiratory distress syndrome
Source: Respir Res. 2023 May 20;24:136. doi: 10.1186/s12931-023-02447-w (PMC10199668; doi:10.1186/s12931-023-02447-w)
Supplement: Supplementary file 1 — Additional file 1. Supplemental methods for targeted metabolomics and quantile g-computation modeling. [file 12931_2023_2447_MOESM1_ESM.docx]

**Additional Material 1**

**Table S1. Time from enrollment to sample processing across groups**

| **Variable** | **Class 1 ARDS**  **(N=50)** | **Class 2 ARDS**  **(N=50)** | **Airway Controls**  **(N=50)** | ***P*-value**  **Class 1 vs. Class 2** | ***P*-value**  **Class 1 vs. Controls** | ***P*-value**  **Class 2 vs. Controls** |
| --- | --- | --- | --- | --- | --- | --- |
| Time from sample collection to metabolomics analysis, median [IQR], years | 5.7 [4.8, 7.3] | 5.9 [4.8, 6.7] | 4.2 [3.2, 5.6] | 0.84 | 0.0005 | 0.0005 |

**Methods**

*Sample processing and metabolite extraction*

Polar metabolite pool extraction for targeted profiling by liquid chromatography high resolution mass spectrometry (LC-HRMS) was performed by adding ice cold 1:1 methanol:ethanol at a ratio of 1:4 sample serum:buffer. Samples were spiked with deuterated (D_3_)-creatinine and (D_3_)-alanine, (D_4_)-taurine, and (D_3_)-lactate (Sigma-Aldrich) as internal standards at a final concentration of 10 µM. After 3 minutes of vortexing, the supernatant was cleared of protein by centrifugation at 16,000 x g and 3 µL of cleared supernatant was subjected to LC-HRMS analysis. For stable isotope dilution analysis of acylcarnitines sample lysates were spiked with the following internal standard mix: 0.76 nmol deuterated (D_9_)-carnitine, 0.19 nmol (D_3_)-acetylcarnitine, 0.038 nmol (D_3_)-propionylcarnitine, 0.038 nmol (D_3_)-butyrylcarnitine, 0.038 nmol (D_9_)-isovalerylcarnitine, 0.038 nmol (D_3_)-octanoylcarnitine, 0.038 nmol (D_9_)-myristoylcarnitine, and 0.038 nmol (D_3_)-palmitoylcarnitine (NSK-B-1, Cambridge Isotopes). 3-methylhistidine was validated against an internal library of purified standards. After 3 minutes of vortexing, the supernatant was cleared of protein by centrifugation at 16,000xg. 2 µL of cleared supernatant was subjected to online LC-HRMS analysis.

*Targeted profiling using liquid chromatography-high resolution mass spectrometry (LC-HRMS)*

For initial screening, targeted profiling analysis of serum was conducted for 25 amino acids along with carnitine and acetylcarnitine. Samples were injected via a Thermo Vanquish ultra-high performance liquid chromatograph (UHPLC) and separated over a reversed phase Thermo HyperCarb porous graphite column (2.1×100mm, 3μm particle size) maintained at 55°C. For the 20-minute LC gradient, the mobile phase consisted of solvent A (water/0.1% formic acid) and solvent B (acetonitrile/0.1% formic acid). The gradient was the following: 0-1 min 1% B, increasing to 15%Bover 5 minutes, followed by an increase to 98% over 5 minutes, and holding at 98% for 5 minutes, followed by equilibration at initial conditions of 1%B for 5 minutes. The Thermo ID-X tribrid mass spectrometer was operated in polarity switching mode (using both positive and negative ionization) in full scan mode (2 μscans) from 100 to 800 *m/z* at 70,000 resolution with an automatic gain control target of 2e5. Source ionization setting was 3.0 kV spray voltage for both positive and negative mode. Source gas parameters were 35 sheath gas, 12 auxiliary gas at 320°C, and 8 sweep gas. Calibration was performed prior to analysis using the PierceTM FlexMix Ion Calibration Solutions (Thermo Fisher Scientific). Integrated peak areas were then extracted manually using XCalibur Quan Browser version 2.7 (Thermo Fisher Scientific) and normalized to ISTD peak area to provide a peak area ratio of analyte to internal standard. The reported metabolite identifications in the targeted profiling analysis are confirmed identifications based on the in-house metabolite library in the mass spectrometry core. A QC pool of all samples was generated and injected every 8 samples to monitor instrument response across the analysis.

*Quantile g-computational modeling for acylcarnitine profiles*

G-computation is a statistical approach which estimates the causal effect from observational data. This multi-step approach builds a series of regression models of outcome against each exposure. Then it uses a counterfactual approach to predicted confounder probability of outcome for each exposure. Final g-computation estimator measures the sum of all regression coefficient of the exposure of interest. Using a marginal structure model and foundation of counterfactual model, it assumes the effect of any given exposure in the final model is adjusted for all exposures. Quantile g-computation (QGC) uses g-computation on quantized exposures. This is the model of choice to take into account potential joint effects of chemicals exposures such as pollutants and metabolomes in which exposures are highly correlated (30-32). Unlike other mixture models such as weighted quantile sum regression, QGC does not require linearity and homogeneity of exposure effect. It calculates a joint exposure measure from the mixture of exposures, and the weight (and direction) of each single exposure on joint exposure measure. It also calculates the effect of increasing this joint exposure by one quantile on risk of outcome by measuring the probability of outcome per each quantile of joint exposure. A plot of this probability versus quantities (and the 95%CI confidence interval) using the “qgcomp” package in R indicates the direction of joint exposure effect on risk of outcome (30-32). Analysis was adjusted for age, sex, and creatinine when comparing Class 1 and Class 2 ARDS subphenotypes within the cohort.

**Acylcarnitines adjusted for age, gender, and creatinine**

Mixture log(RR) (bootstrap CI):

$coefficents

Estimate Std. Error Lower CI Upper CI Z value Pr(>|z|)

(Intercept) -1.0964302 0.2979347 -1.68037143 -0.5124890 -3.680103 0.0002331398

psi1 0.2388032 0.1335533 -0.02295641 0.5005627 1.788074 0.0737640040

Call:

bayesglm(formula = f, family = ..1, data = qdata[, nidx, drop = FALSE],

weights = weights)

Deviance Residuals:

Min 1Q Median 3Q Max

-1.60511 -0.92903 0.05418 0.94772 1.96140

Coefficients:

Estimate Std. Error z value Pr(>|z|)

(Intercept) -0.76660 0.51108 -1.500 0.13363

carnitine -0.34121 0.33020 -1.033 0.30144

acetylcarnitine 0.45023 0.31925 1.410 0.15846

propionylcarnitine -0.11839 0.38894 -0.304 0.76083

butylcarnitine -0.25143 0.34091 -0.738 0.46081

isovalerylcarnitine 0.05322 0.33957 0.157 0.87547

octanoylcarnitine 0.93457 0.32233 2.899 0.00374 **

myristoylcarnitine -0.23936 0.38221 -0.626 0.53115

palmitoylcarnitine 0.01027 0.35993 0.029 0.97723

---

Signif. codes: 0 ‘***’ 0.001 ‘**’ 0.01 ‘*’ 0.05 ‘.’ 0.1 ‘ ’ 1

(Dispersion parameter for binomial family taken to be 1)

Null deviance: 121.99 on 87 degrees of freedom

Residual deviance: 100.49 on 79 degrees of freedom

AIC: 118.49

Number of Fisher Scoring iterations: 7

quantile quantile.midpoint linpred rr se.lnrr ll.rr ul.rr ll.linpred ul.linpred

0 0 0.125 -1.0964302 1.000000 0.0000000 1.0000000 1.000000 -1.096430 -1.09643022

1 1 0.375 -0.8576271 1.269729 0.1335533 0.9773051 1.649649 -1.119387 -0.59586748

2 2 0.625 -0.6188239 1.612211 0.2671065 0.9551252 2.721343 -1.142343 -0.09530473

3 3 0.875 -0.3800207 2.047070 0.4006598 0.9334487 4.489262 -1.165299 0.40525801
