# Supplementary material for: Serum metabolomic signatures of fatty acid oxidation defects differentiate host-response subphenotypes of acute respiratory distress syndrome
Source: Respir Res. 2023 May 20;24:136. doi: 10.1186/s12931-023-02447-w (PMC10199668; doi:10.1186/s12931-023-02447-w)
Supplement: Supplementary file 2 — Additional file 2. Supplemental data including biomarker profiles and additional acylcarnitine analyses. [file 12931_2023_2447_MOESM2_ESM.pdf]

Additional Data 2
Figure S1

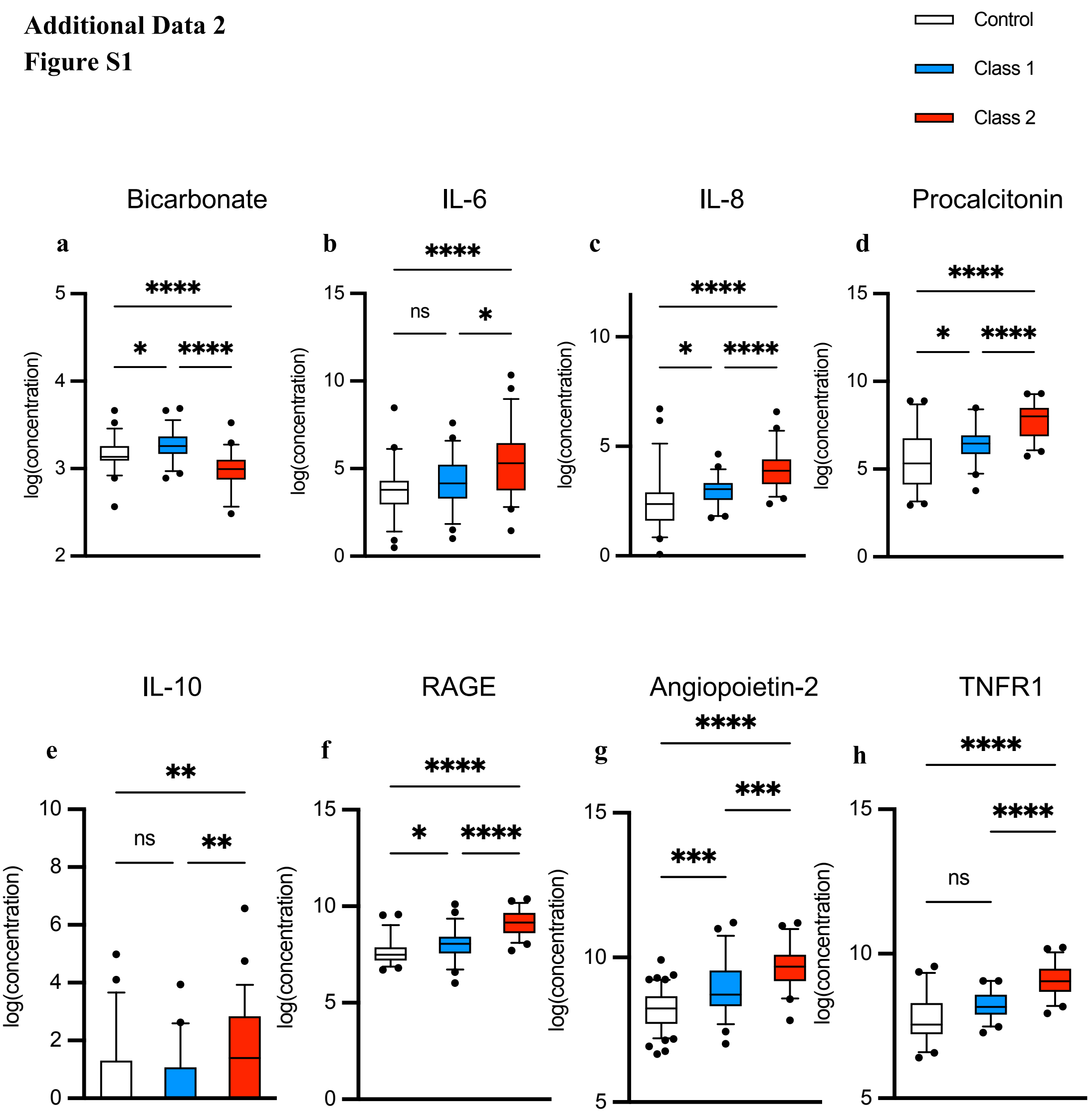

Figure S2

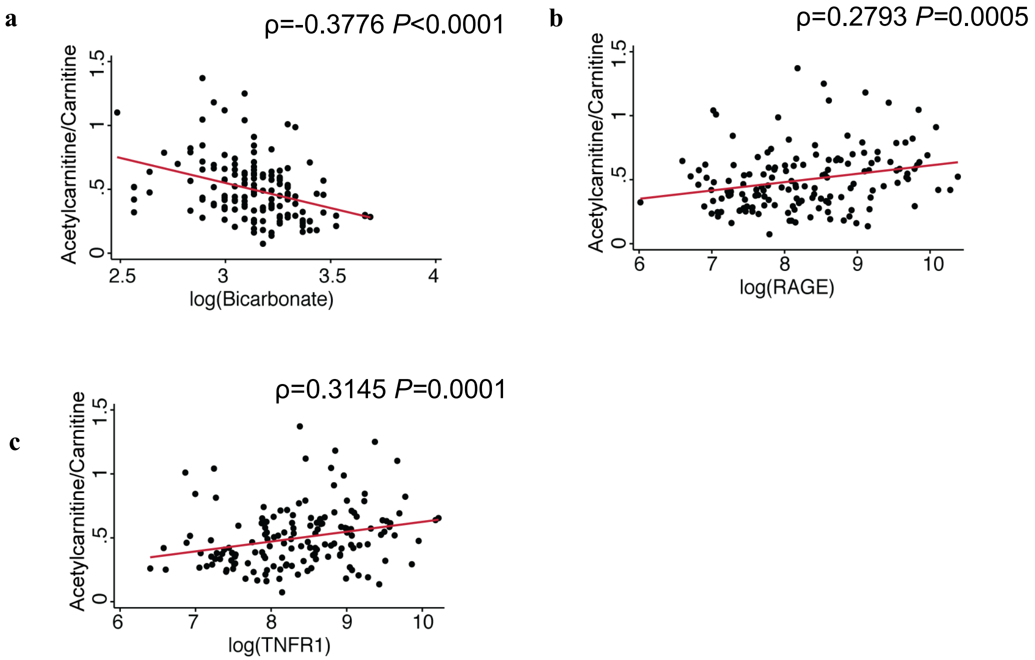
a
ρ=-0.3776 P<0.0001
Acetylcarnitine/Carnitine
0
.5
1
1.5
2.5
3
3.5
4
log(Bicarbonate)
b
ρ=0.2793 P=0.0005
Acetylcarnitine/Carnitine
0
.5
1
1.5
6
7
8
9
10
log(RAGE)
c
ρ=0.3145 P=0.0001
Acetylcarnitine/Carnitine
0
.5
1
1.5
6
7
8
9
10
log(TNFR1)

Figure S3

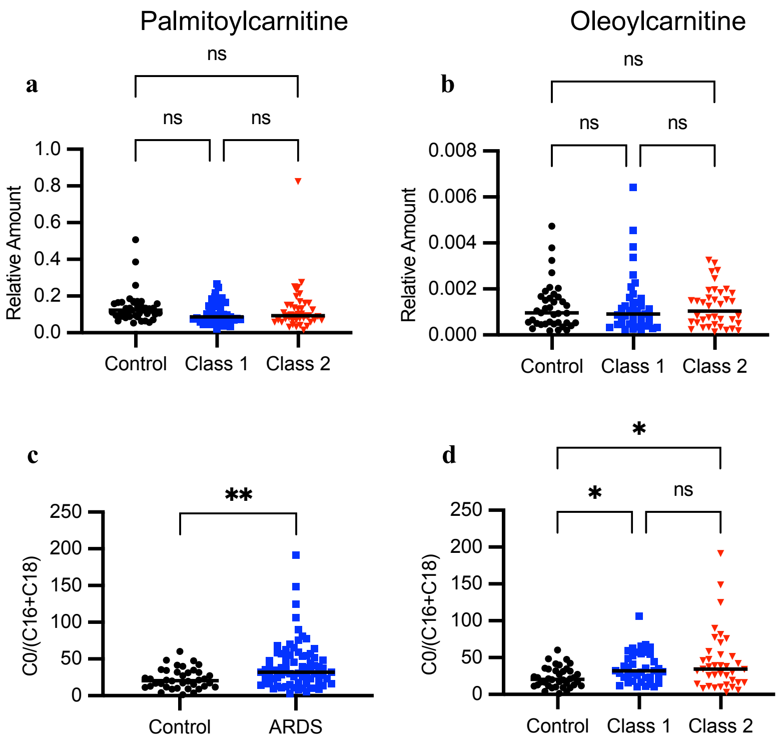

Palmitoylcarnitine
Oleoylcarnitine
a
b
c
d
ns
Relative Amount
1.0
0.8
0.6
0.4
0.2
0.0
0.008
0.006
0.004
0.002
0.000
Control
Class 1
Class 2
ARDS
C0/(C16+C18)
250
200
150
100
50
0
**
*

Figure S1. Biomarkers profiles within cohort of airway controls, Class 1, and Class 2 ARDS groups. Serum biomarkers were measured by a customized Luminex immunoassay (R&D Systems). Log transformation of values is shown. Box and whisker plots depict median 50% of values as box with mean denoted by straight line and whiskers showing 5th to 95th percentile values with outliers denoted as black dots. Panels include the log values of the following: (**a**) bicarbonate (mEq/mL)), (**b**) IL-6 (pg/mL), (**c**) IL-8 (pg/mL), (**d**) IL-10 (pg/mL), (**e**) RAGE (pg/mL), (**f**) angiopoietin-2 (pg/mL), (**g**) TNFR1 (pg/mL), and (**h**) procalcitonin (pg/mL). The white bars represent control patients (N=50) while the blue and red bars represent Class 1 (N=50) and Class 2 (N=50) groups, respectively. Group comparisons were performed using the Kruskal-Wallis test. Asterisks indicate $P<0.05$ (*), $P<0.01$ (**), $P<0.001$ (***), $P<0.0001$(****), and ns (not significant).

Figure S2. Correlations of acetylcarnitine/carnitine ratio with serum biomarkers. Pairwise correlations are shown for acetylcarnitine:carnitine ratio and (**a**) log(bicarbonate), (**b**) log(RAGE), and (**c**) log(TNFR1) values. Spearman's coefficient (ρ) and *P*-values are shown.

Figure S3. Serum acylcarnitines as an estimate of relative carnitine palmitoyl transferase activity does not differ between ARDS classes. Controls are shown as black circles (N=42), Class 1 as blue squares (N=44), and Class 2 as red triangles (N=43). Relative amounts of palmitoylcarnitine and oleoylcarnitine are shown (**a** and **b**). The ratio of free carnitine to palmitoylcarnitine plus oleoylcarnitine was calculated from mass spectrometry data as a screening test for carnitine palmitoyl transferase (CPT1) activity (28). Group comparisons were performed using the Kruskal-Wallis test. Black bars represent the median and asterisks denote * $P<0.05$, ** $P<0.01$, and ns (not significant).
